# Comparison of non-linear models for growth characterization of purebred Ayrshire and crossbred cattle

**DOI:** 10.5194/aab-68-721-2025

**Published:** 2025-12-09

**Authors:** Mykhailo Matvieiev, Ayhan Ceyhan, Özge Kozaklı, Andriy Getya, Oleksandr O. Borshch, Sergiy Ruban

**Affiliations:** 1 Department of Technologies in Animal Husbandry, National University of Life and Environmental Sciences of Ukraine, Kyiv, 03041, Ukraine; 2 Department of Applied Biology, Animal Breeding and Genetics, National University of Life and Environmental Sciences of Ukraine, Kyiv, 03041, Ukraine; 3 Department of Animal Production and Technologies, Niğde Ömer Halisdemir University, Niğde, 51240, Türkiye; 4 Department of Technology of milk and meat production, Faculty of Biotechnological, Bila Tserkva National Agrarian University, Bila Tserkva, 09117, Ukraine; 5 The National Academy of Agrarian Sciences of Ukraine, Kyiv, 03041, Ukraine; 6 Department of Animal Productivity Biology named after O.V.Kvasnytskyi, Poltava State Agrarian University, Poltava, 36003, Ukraine

## Abstract

The objective of this study was to compare four non-linear mathematical models in terms of evaluating the growth pattern of Ayrshire and crossbred (Norwegian Red × Ayrshire) cattle. Monthly live-weight (kg) records of 725 cows were collected from birth to 540 d of age. The growth curves were analyzed using negative exponential, logistic, Richard, and Gompertz models. Parameters were estimated with the NLMIXED procedure, and model fit was assessed using Akaike's information criterion (AIC), the Bayesian information criterion (BIC), the overall standard error (OSE), and the adjusted coefficient of determination (Adj- $R^{2}$ ). The Gompertz model occasionally provided higher explanatory power (Adj- $R^{2}$ up to 0.95) and lower prediction error (OSE as low as 4.5), but the Richard model consistently yielded the lowest AIC and BIC values, making it the most reliable and parsimonious choice. Growth curves showed that females attained substantially higher mature body weights than males, with crossbred cattle generally being heavier than purebred Ayrshire. These findings demonstrate the utility of the Richard model for accurately describing and predicting cattle growth, providing valuable information for genetic selection, herd management, and breeding strategies.

## 1 Introduction

Livestock production in Ukraine plays an important role in the country's agricultural economy (Stavetska et al., 2022), providing the population with significant quantities of milk (Bal-Prylypko et al., 2024; Ruban et al., 2022) and meat (Antoniv et al., 2023). According to the State Statistics Service of Ukraine, at the beginning of 2023, there were 1 352 800 cows of various breeds on all categories of farms in Ukraine (SSSU, 2024).

Intensive increase in weight and body size, commonly referred to as growth, is an important fundamental goal of animal breeding (Kozakli et al., 2022; Kramarenko et al., 2022). Scientists have described growth in three stages, stating that growth starts at a certain point and gradually increases in the first stage, which is characterized by a partially linear shape of the growth curve until it reaches the inflection point in the second stage, and, finally, in the third stage, the growth curve approaches an asymptote (Waheed et al., 2011). Ghavi Hossein-Zadeh (2017) emphasized the importance of observing the growth of animals throughout their lives, while Sakar et al. (2023) emphasized the importance of determining the optimal body weight, breeding age, slaughter age, and feeding procedures. Budimulyati et al. (2012) stated that the age of sexual maturity for first mating in cattle is determined by the optimum body weight (Sakar et al., 2023). From an economic point of view, it is essential to produce more meat in a shorter feeding period (Domínguez-Viveros et al., 2023). In addition, Ismirandy (2018) recommended that growth curves be used to select breeding strategies for high-producing animals, taking into account their growth performance. Gemuh (2020) highlighted that calves continue to grow optimally between 1–8 months. For example, the value of cattle at slaughter is highly dependent on the amount of muscle in the carcass. Body weight is strongly related to other economically important traits, including production and reproductive traits (Abdallah and McDaniel, 2000). In order to maintain the high productivity of cattle, optimization of genetic improvement programs is important (Matvieiev et al., 2023). Body weight gain after weaning in cattle represents the growth potential of the animal, which is an important trait in animal selection (Raungprim et al., 2023). A significant advantage of some breeds in this respect may lead to a decision to change the breed composition of the herd (Christensen et al., 1984). It should be noted that mating cows of Ukrainian origin with the semen of foreign sires allows one not only to increase growth energy but also to increase milk production and to reduce the percentage of stillborn calves (Bashchenko et al., 2023; Borshch et al., 2021).

Of particular interest is the experience with the Ayrshire and Norwegian Red breeds. Ayrshire crosses are known to have higher growth energy than the parent breeds (Robison et al., 1980). In addition, such crossbred animals have higher milk production. In particular, the milk yield of primiparous (F_1_) cows obtained by crossbreeding Finnish Ayrshire and Danish Red was 859 and 1045 kg higher than that of purebred cows of the corresponding parent breeds (Pedersen and Christensen, 1989). In Croatia, first-generation crosses of Holstein and Norwegian Red were characterized by higher milk yield, better milk composition (fat, protein), and shorter days open than pure Holsteins (Benak et al., 2020). In addition to the increase in milk productivity, the Norwegian Red × Ayrshire also showed an improvement in milk nutrition values (Ruban et al., 2023).

In addition, reaching optimal weight by a certain age is an important goal for many breeding programs (Halvoník et al., 2023). It is also important to note that environmental factors should be taken into account when assessing the growth curve (Mokhtari et al., 2023), which can be done using individual statistical models (Weigel et al., 2017). There are various non-linear mixed mathematical models such as the Brody, von Bertalanffy, logistic, Gompertz, Richards, monomolecular, and Weibull models (Beltran et al., 1992; Waiz et al., 2019) that are used to describe growth curves.

The main objective of this study was to compare four different non-linear mathematical models used to characterize the growth pattern of Ayrshire and crossbred cattle of both sexes.

## 2 Materials and methods

### 2.1 Study location

The research was conducted on a commercial farm in Poltava oblast ( $50{}^{\circ}02^{\prime }39^{\prime \prime }$ N, $33{}^{\circ}51^{\prime }09^{\prime \prime }$ E) in Ukraine.

### 2.2 Animal material

The animal material for the study consisted of male and female Ayrshire and crossbred F_1_ (Norwegian Red × Ayrshire) cattle.

### 2.3 Animal feeding and management

In the first 6 h after birth, the calves received colostrum up to 10 % of their body weight. The calves were then fed 8–10 L of whole row milk per day in three shifts for 30 d using the URBAN milk shuttle with a milk heater. During this period, the calves were housed in individual pens. If the calves were healthy, they were moved to group housing (20 animals per group) at 30–35 d of age. In the group section, the calves received a milk replacer through the URBAN PAULA robotic feeder. The milk replacer was fed at a rate of 8 $L\;d^{-1}$ four times a day. Throughout the milk-feeding period, which lasts until the animals reach a live weight of 90–100 kg, the calves consume up to 35 kg of pelleted feed, 250–300 L of whole milk, and 220–250 L of milk replacer. At the end of the milk-feeding period, the calves are moved to a section where they receive compound feed (up to 2 kg per head per day) and unlimited hay and silage. The amount of dry matter (DM) in the diet and the concentration of nutrients in the diet changed with the age of the animals. From 2 months of age to the establishment of fertility, the amount of DM in the daily diet increased from 2.8 to 10.8 kg, and the metabolic energy content per 1 kg of DM decreased from 11.8 to 9.0 MJ of energy. Crude protein concentration ranged from 18 % to 13 % in the diets of younger and older animals, respectively.

**Table 1:** Non-linear models evaluated for Ayrshire and crossbred F_1_ cattle.

Model	Equation		References
Negative exp.	$y_{i}=\beta_{0}\cdot (1-\mathrm{exp}(-\beta_{2}\cdot \text{day}))+e_{i}$	(1)	Chen (2000)
Logistic	$y_{i}=\beta_{0}/(1+\mathrm{exp}(-(\beta_{1}\cdot \text{day}+\beta_{2})))$	(2)	Nelder (1961)
Richard	$y_{i}=\beta_{0}/(1+\beta_{1}\cdot \mathrm{exp}(-\beta_{2}\cdot \text{day}))**(1/\beta_{3})+e_{i}$	(3)	Richard (1959)
Gompertz	$y_{i}=\beta_{0}\cdot \mathrm{exp}(-\beta_{1}\cdot \mathrm{exp}(-\beta_{2}\cdot \text{day}))+e_{i}$	(4)	Gompertz (1825)

### 2.4 Growth performance

In this study, the database consisted of monthly live weight measurements at intervals from birth to 540 d of age. The live weights of the animals were measured monthly from birth until the age of first mating (for females) and until the age of sale (for males) using a weighing machine. Sex and genotype information was also recorded. This study used information from a total of 443 animals (60 males and 343 females) of the Ayrshire breed and 282 animals (117 males and 165 females) of crossbred (F_1_) genotypes born in 2018 and 2021.

### 2.5 Statistics analysis

In the study, four different non-linear models were used to estimate the growth curves: negative exponential, logistic, Richard, and Gompertz (Table 1).

In the model equations, $y_{i}$ denotes live weight (in kg), measured on day t ; $\beta_{0}$ denotes the asymptotic value; $\beta_{1}$ denotes the integration constant; $\beta_{2}$ denotes the slope of the growth curve value considered to be best fitted; and $\beta_{3}$ denotes the model shape and slope control parameter (Aggrey, 2002; Kizilkaya et al., 2006; Soysal et al., 2001; Yıldız et al., 2009).

Analyses were performed for each sex using the dual quasi-Newton method of the NLMIXED procedure of the SAS statistical analysis software. Each subgroup model (sex × breed) was fitted separately, and, within each model, the significance of the estimated parameters was evaluated ( P<0.05 ). Graphical visualization was used. Specifically, the SGPlot procedure in SAS was used for visualization.

The selection of the best-fitting model was based on the following criteria: Akaike's information criterion (AIC), the Bayesian information criterion (BIC), the coefficient of determination ( $R^{2}$ ), the overall standard error (OSE), and the adjusted coefficient of determination (Adj- $R^{2}$ ), calculated according to the following mathematical formulae:

5R2=1-SSETSS,6Adj-R2=1-((1-R2)⋅(n-1)/(n-k-1)),7OSE=(SSE/(n-p-1)),8AIC=n×nlSSEn+2k,9BIC=n×nlSSEn+k⋅nl(n),

where Adj- $R^{2}$ refers to the adjusted coefficient of determination (Adj- $R^{2}$ ), $R^{2}$ refers to the coefficient of determination, SSE refers to the sum of the square error, TSS refers to the total sum of square, OSE refers to the overall standard error, AIC refers to Akaike's information criterion, BIC refers to the Bayesian information criterion, n refers to the total number of data, k refers to the number of parameters in the model, and nl refers to the natural logarithm (Domínguez-Viveros et al., 2023).

## 3 Results

Mean body weights, standard deviations, and coefficients of variation were calculated for different ages by sex (Tables 2–5).

**Table 2:** The descriptive statistics of live weight of male Ayrshire cattle at different ages.

Ages	n	Mean	SD	Min.	Max.	CV
(in days)		(kg)		(kg)	(kg)	(%)
Birth weight	60	29.78	0.72	28.00	31.00	2.40
30	39	50.95	13.49	30.00	71.00	26.48
60	60	71.60	11.97	53.00	101.00	16.72
90	60	91.57	14.22	54.00	137.00	15.53
120	60	114.48	19.94	77.00	178.00	17.41
150	57	140.49	21.02	104.00	198.00	14.96
180	41	166.54	25.65	123.00	218.00	15.40
210	29	198.41	21.94	142.00	241.00	11.06
240	18	216.28	23.10	178.00	258.00	10.68
270	14	236.00	26.18	177.00	268.00	11.09
300	2	218.50	30.41	197.00	240.00	13.92
330	2	265.00	29.70	244.00	286.00	11.21

N : number of animals; SD: standard deviation; CV: coefficient of variation; min.: minimum; max.: maximum.

**Table 3:** The descriptive statistics of live weight of female Ayrshire cattle at different ages.

Ages	n	Mean	SD	Min.	Max.	CV
(in days)		(kg)		(kg)	(kg)	(%)
Birth weight	383	29.38	1.36	25.00	48.00	4.63
30	351	47.79	10.56	29.00	94.66	22.10
60	359	72.87	12.94	28.87	123.16	17.76
90	371	93.34	13.67	60.00	180.51	14.65
120	372	112.02	16.43	80.00	168.89	14.66
150	379	132.11	19.65	87.00	202.00	14.87
180	340	154.24	21.32	94.38	220.00	13.82
210	341	174.57	23.61	95.92	255.00	13.53
240	349	197.38	24.31	137.00	277.00	12.32
270	323	218.20	27.03	138.00	300.00	12.39
300	308	241.92	27.96	160.00	335.00	11.56
330	317	265.98	30.87	181.00	389.66	11.61
360	329	291.57	32.58	185.00	412.00	11.17
390	332	315.85	34.17	202.00	430.00	10.82
420	314	338.62	36.21	129.69	449.00	10.69
450	291	361.91	35.96	192.31	464.00	9.94
480	273	385.20	34.14	224.56	479.00	8.86
510	256	406.26	31.84	267.98	494.00	7.84
540	242	428.93	29.29	335.62	510.00	6.83

N : number of animals; SD: standard deviation; CV: coefficient of variation; min.: minimum; max.: maximum.

**Table 4:** The descriptive statistics of live weight of male crossbred (F_1_) cattle at different ages.

Ages	n	Mean	SD	Min.	Max.	CV
(in days)		(kg)		(kg)	(kg)	(%)
Birth weight	117	29.66	0.56	28.00	31.00	1.89
30	80	48.15	12.55	30.00	77.00	26.07
60	117	69.74	15.01	35.00	109.00	21.52
90	117	93.64	18.88	50.00	138.00	20.16
120	117	113.93	19.52	74.00	173.00	17.13
150	116	134.30	22.01	84.00	192.00	16.39
180	115	158.89	25.05	98.00	216.00	15.77
210	94	190.15	24.71	132.00	250.00	12.99
240	60	210.73	25.28	153.00	276.00	12.00
270	40	229.10	45.24	22.00	303.00	19.75
300	17	244.00	34.05	190.00	312.00	13.95
330	11	254.00	13.56	234.00	270.00	5.34

N : number of animals; SD: standard deviation; CV: coefficient of variation; min.: minimum; max.: maximum.

**Table 5:** The descriptive statistics of live weight of female crossbred (F_1_) cattle at different ages.

Ages	n	Mean	SD	Min.	Max.	CV
(in days)		(kg)		(kg)	(kg)	(%)
Birth weight	165	29.32	1.41	28.00	45.00	4.79
30	150	52.96	14.28	29.00	92.74	26.97
60	162	81.88	14.12	49.00	115.57	17.24
90	164	101.11	13.32	71.00	148.33	13.18
120	165	118.34	18.91	42.23	165.00	15.98
150	165	144.14	19.33	90.00	211.26	13.41
180	164	166.84	21.53	107.30	240.51	12.90
210	159	191.57	22.19	129.59	260.26	11.58
240	147	217.65	28.99	152.72	392.59	13.32
270	135	243.12	32.98	173.00	393.84	13.57
300	126	264.07	29.79	203.00	350.00	11.28
330	116	289.12	38.43	212.00	453.84	13.29
360	102	310.35	38.25	241.00	456.30	12.33
390	93	331.94	33.95	270.00	408.00	10.23
420	80	346.74	64.10	131.28	427.00	18.49
450	72	373.41	32.06	301.00	442.00	8.59
480	53	392.79	32.57	334.00	460.00	8.29
510	44	416.81	29.61	365.00	477.00	7.10
540	43	437.47	27.91	384.00	492.00	6.38

N : number of animals; SD: standard deviation; CV: coefficient of variation; min.: minimum; max.: maximum.

The birth weights of Ayrshire and crossbred calves at birth were 29.78 and 29.66 kg for males and 29.38 and 29.32 kg for females. The 330 d live weights of Ayrshire and crossbred animals were 265.00 and 289.12 kg for males and 265.98 and 289.12 kg for females, respectively. When the differences in body weight were analyzed, the males had a higher body weight than the females. Meanwhile, the crossbred genotype had a higher body weight than Ayrshire cattle.

Considering Adj- $R^{2}$ , OSE, AIC, and BIC as performance criteria, model selection is based primarily on the information criteria (AIC and BIC). On this basis, across both sexes, as shown in Table 7, the Richard model provides the best overall fit. By contrast, the Gompertz model often attains higher explanatory power (greater Adj- $R^{2}$ ) and lower prediction error (smaller OSE). Because AIC and BIC penalize model complexity and offer a principled basis for comparative selection, the Richard model is retained as the preferred specification, while the Gompertz model's strengths in terms of Adj- $R^{2}$ and OSE are acknowledged (Table 6).

**Table 6:** The goodness-of-fit criteria for the studied growth models.

Genotype	Sex	Model	Adj- $R^{2}$	OSE	AIC	BIC
Ayrshire	Female	Negative exponential	0.9265	33.29	10 675 626.85	10 675 647.06
		Logistic	0.9265	27.69	7 744 223.46	7 744 250.41
		Gompertz	0.9492	26.60	1 357 455.97	1 357 482.92
		Richard	0.9531	33.70	1 289 036.86	1 289 070.54
	Male	Negative exponential	0.9247	22.22	263 998.34	264 010.61
		Logistic	0.8699	21.17	218 827.85	218 844.22
		Gompertz	0.8819	17.94	187 557.43	187 573.80
		Richard	0.9152	19.26	6114.87	6135.32
Crossbred	Female	Negative exponential	0.9023	30.33	2 855 033.53	2 855 050.76
		Logistic	0.9301	29.08	2 479 312.87	2 479 335.84
		Gompertz	0.9357	29.07	2 393 954.52	2 393 977.49
		Richard	0.9358	31.49	510 943.50	510 972.21
	Male	Negative exponential	0.9246	28.34	806 470.54	806 485.27
		Logistic	0.8125	21.96	594 387.97	594 407.61
		Gompertz	0.8874	21.44	424 249.19	424 268.83
		Richard	0.8927	22.19	22 786.35	22 810.90

**Figure 1:**
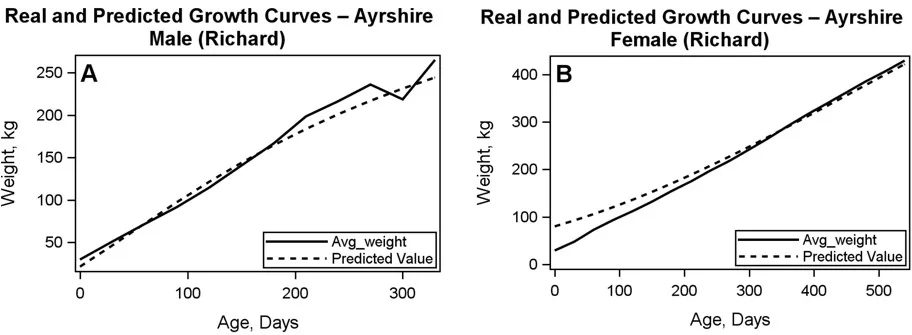
The growth curve of body weight in Ayrshire cattle. **(A)** Male, **(B)** female.

It should be noted that Adj- $R^{2}$ values were high for all models describing Ayrshire growth, ranging from 0.8699 to 0.9531. Based on AIC and BIC, the Richard model provided the best overall fit for Ayrshire cattle for both males and females. The body weight growth curves (observed and Richard-predicted) for Ayrshire cattle are shown in Fig. 1 (A: male; B: female).

**Figure 2:**
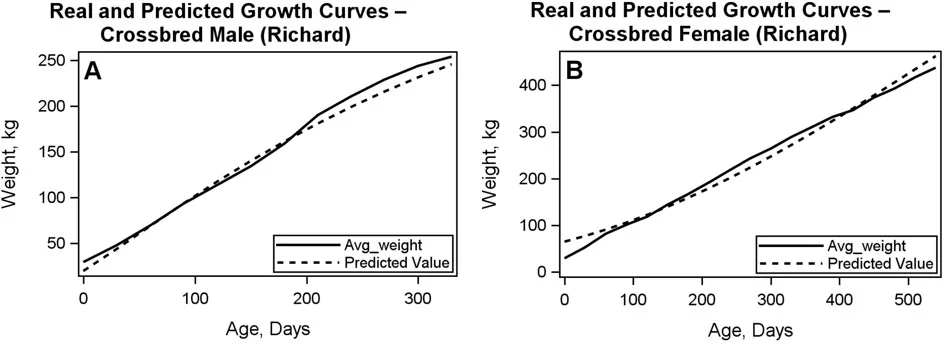
The growth curve of body weight in crossbreed cattle. **(A)** Male, **(B)** female.

For the models describing the growth of crossbred (F1) cattle, the adjusted $R^{2}$ values ranged from 0.8125 to 0.9358. Because model selection prioritized AIC and BIC, the Richard model provided the best fit for both male and female crossbred (F1) cattle, yielding the lowest AIC and BIC values (Table 6). The body weight growth curves of crossbred (F1) cattle were plotted using the Richard model and are presented in Fig. 2.

The growth parameters for Ayrshire and crossbred F_1_ cattle are presented in Table 7.

**Table 7:** Regression coefficients and growth curve parameters for body weight of Ayrshire cattle and crossbred cattle (F_1_). A dot (.) denotes that a parameter is not included in the model structure. These parameters are not missing or undefined; they are simply not part of the functional form of the respective nonlinear model. Therefore no estimate exists by design.

Genotype	Sex	Model	$\beta_{0}$	$\beta_{1}$	$\beta_{2}$	$\beta_{3}$	$S_{e}^{2}$
Ayrshire	Female	Negative exponential	724.42	.	0.001355	.	0.6469
		Gompertz	559.77	2.8336	0.004142	.	3.2927
		Logistic	501.45	8.4151	0.006703	.	0.6173
		Richard	966.00	0.1669	0.002132	0.06200	5.5800
	Male	Negative exponential	365.39	.	0.003417	.	0.8253
		Gompertz	339.84	2.4422	0.006871	.	0.7563
		Logistic	466.98	9.0605	0.008176	.	0.9025
		Richard	356.50	-0.8762	0.003861	-0.7434	45.0315
Crossbred	Female	Negative exponential	669.77	.	0.001716	.	0.7427
		Gompertz	558.30	2.5113	0.003877	.	0.8137
		Logistic	455.23	7.1577	0.007568	.	0.7862
		Richard	1371.22	-0.09056	0.001855	-0.03112	4.5341
	Male	Negative exponential	260.91	.	0.004831	.	0.9971
		Gompertz	342.85	2.3422	0.006294	.	1.0859
		Logistic	247.82	6.0980	0.01392	.	0.8122
		Richard	388.34	-0.8970	0.003362	-0.7658	27.8932

The regression coefficients are $\beta_{0}$ , $\beta_{1}$ , and $\beta_{2}$ , where $\beta_{0}$ is the asymptotic value, interpreted as the adult weight parameter; $\beta_{1}$ is an adjustment parameter; and $\beta_{2}$ is the growth rate, expressing weight gain as a proportion of total weight. The standard error $S_{e}^{2}$ indicates the level of uncertainty in predictions.

The prediction of the Richard model for female Ayrshire cattle is as follows.

The data obtained were used to fit regression equations to predict weight.

10 $$\begin{array}{rl} & y_{i}=\\ & 966/((1-0.1669\cdot \mathrm{exp}(-0.002132\cdot \text{day}))**(1/(-0.062))) \end{array}$$

In the Richards model equation, the double asterisk ( ∗∗ ) denotes exponentiation (raising to a power). For example, X∗∗ (1/ $\beta_{3}$ ) means X raised to the power of (1/ $\beta_{3}$ ). The asymptotic value ( $\beta_{0}$ ) of 966 kg is the maximum weight that purebred Ayrshire females can reach as they grow older. It represents the highest body weight they can achieve over time. $\beta_{1}=-0.1669$ shows how the growth rate changes over time. In this case, the negative value indicates a decreasing growth rate; i.e., weight gain slows down as Ayrshire females mature. The slope of the growth rate curve ( $\beta_{2}$ ) is 0.002132, reflecting the rate at which the growth curve rises. A higher value indicates a steeper growth curve, implying faster weight gain during certain growth periods. The parameter ( $\beta_{3}$ ), with a value of -0.062 , influences the shape and slope of the growth curve. A negative value of ( $\beta_{3}$ ) results in a concave growth curve, where the growth rate decreases over time and approaches a plateau. The error variance ( $S_{e}^{2}$ ) of 5.5800 represents the variability or spread of actual live weights around the values predicted by the model. A lower $S_{e}^{2}$ indicates a better fit of the model to the observed data, with less variability in the predicted weights.

The prediction of the Richard model for female F1 crossbred cattle is as follows:

11 $$\begin{array}{rl} & y_{i}=\\ & 1371.22/((1-0.09056\cdot \mathrm{exp}(-0.001855\cdot \text{day}))\cdot (1/(-0.03112))). \end{array}$$

The asymptotic value ( $\beta_{0}$ ) of 1371.22 kg is the maximum weight that female crossbred F_1_ cattle can reach as they grow older. It represents the highest weight they can reach over time. $\beta_{1}=-0.09056$ indicates how the growth rate changes over time. In this case, the negative value indicates a decreasing growth rate, which means that weight gain slows down as the crossbred F_1_ cattle mature. The slope of the growth rate curve ( $\beta_{2}$ ) is 0.001855, reflecting the rate at which the growth curve rises. A higher value indicates a steeper growth curve, implying faster weight gain during specific periods of growth. The parameter $\beta_{3}$ , with a value of -0.03112 , influences the shape and slope of the growth curve. A negative value of $\beta_{3}$ results in a concave growth curve, where the growth rate decreases over time and approaches a plateau. The error variance ( $S_{e}^{2}$ ) of 4.5341 represents the variability or scatter of actual live weights around the values predicted by the model. A lower $S_{e}^{2}$ indicates a better fit of the model to the observed data, with less variability in the predicted weights.

The prediction of the Richard model for male Ayrshire cattle is as follows:

12 $$\begin{array}{rl} & y_{i}=\\ & 356.5/((1-0.8762\cdot \mathrm{exp}(-0.003861\cdot \text{day}))\cdot (1/(-0.7434))). \end{array}$$

Here, $\beta_{0}=356.5$ kg is the asymptotic (mature) body weight that males approach with age; it represents the maximum attainable weight over time. The adjustment parameter $\beta_{1}=0.8762$ is linked to the initial acceleration of growth; its negative sign implies that early gains are relatively fast but progressively decelerate as animals mature. The growth rate parameter $\beta_{2}=0.003861$ governs how quickly weight increases per unit time; larger values would imply a steeper rise and an earlier approach to maturity. The shape parameter $\beta_{3}=-0.7434$ determines the curvature of the trajectory; a negative value yields a sigmoidal curve with clear deceleration toward a plateau. The model error variance $S_{e}^{2}=45.0315$ quantifies the dispersion of observed weights around fitted values.

The Richard model prediction for crossbred F_1_ male cattle is as follows:

13 $$\begin{array}{rl} & y_{i}=\\ & 388.34/((1-0.8970\cdot \mathrm{exp}(-0.003362\cdot \text{day}))\cdot (1/(-0.7658))). \end{array}$$

In this equation, $\beta_{0}=388.34$ kg denotes the mature (asymptotic) weight. The adjustment parameter $\beta_{1}=-0.8970$ modulates the early phase of growth; its negative sign indicates rapid early gain followed by tapering. The growth rate parameter $\beta_{2}=0.003362$ controls the speed at which body weight increases; higher values correspond to a steeper rise. The shape parameter $\beta_{3}=-0.7658$ governs the curvature and the approach to the plateau. The error variance $S_{e}^{2}=27.8932$ reflects the residual variability around the fitted curve.

## 4 Discussion

In this study, only appropriate models were selected for the description of the growth curve. The Gompertz model provided the best fit of the growth curve for male Ayrshire and crossbred cattle, and the Richard model provided the best fit for female Ayrshire and crossbred cattle due to higher Adj- $R^{2}$ and lower OSS, AIC, and BIC than other models. The results of the present study are in agreement with the reports of Hartati and Putra (2021), who evaluated the growth curves in male and female Madura cattle using logistic, Gompertz, and von Bertalanffy functions due to the simplicity of their interpretation. Similarly, Inoue et al. (2020) determined the growth curve and reported that the Gompertz model gave the best fit in Japanese Black cattle. Similarly, Tutkun (2019) evaluated the Gompertz, Richard, and logistic models and selected the Richard model as that which best fitted the equation for Holstein-Friesian bulls. Júnior et al. (2022) compared six different growth models (Brody, Gompertz, logistics, Richard, Meloun, von Bertalanfy) describing the growth curve of Nelore cattle and found that the Brody function gave the best-fitting results. In addition, Adinata et al. (2022) described growth patterns in Ongole grade cattle and found that the Brody model gave the best fit to the growth curve. Meanwhile Sakar et al. (2023) compared six different non-linear models to describe the growth curve of animals (second-degree polynomial, third-degree polynomial, logistic, Brody, von Bertalanffy, Gompertz), and, according to the results obtained, the third-degree polynomial model was the best to describe the growth curve of Anatolian Black cattle.

Hartati and Putra (2021) calculated $\beta_{0}$ in Madura cattle using the logistic, Gompertz, and von Bertalanffy models and obtained values of 218.02, 274.13, and 329.83 kg for females and 220.80, 277.72, and 333.92 kg for males. Júnior et al. (2022) determined $\beta_{0}$ values of 357.51 and 334.00 kg for male and 306.93 and 289.00 kg for female Nelore cattle using the Gompertz and logistic models. Domínguez-Viveros et al. (2023) evaluated $\beta_{0}$ using the Gompertz, logistic, and Brody models, obtaining values of 491.0, 408.2, and 1645.9 kg for males and 715.1, 402.9, and 352.3 kg for females in Limousin cattle. Tutkun (2019) estimated $\beta_{0}$ in Holstein-Friesian bulls using Gompertz, Richards, and logistic models, obtaining 986.440, 1110.24, and 672.940 kg. Sakar et al. (2023) calculated $\beta_{0}$ in Anatolian Black cattle using different models: logistic – 206.09 kg, Brody – 225.56 kg, and Gompertz – 129.05 kg. Budimulyati et al. (2012) found that 29- and 21-month-old Holstein-Friesian heifers had $\beta_{0}$ values of 343.6 and 306.3 kg (logistic model) and 354.5 and 319.1 kg (Gompertz model). Adinata et al. (2022) calculated $\beta_{0}$ in Ongole grade cattle using Brody, logistic, and Gompertz models and obtained 857.639, 596.868, and 640.136 kg for males and 692.346, 04.237, and 537.936 kg for females, respectively. The $\beta_{0}$ was also calculated using logistic, Gompertz, and Brody models for Swamp buffalo (*Bubalus b. carabanensis*): 675.10, 698.18, and 902.00 kg (Raungprim et al., 2023). On the other hand, the $\beta_{0}$ values obtained using different models for some breeds of *Bos indicus* cattle were similar to those of Domínguez-Viveros et al. (2023) for Limousin cattle but did not agree with the results of Adinata et al. (2022) for Ongole grade cattle, Tutkun (2019) for Holstein-Friesian bulls, and Sakar et al. (2023) for Anatolian Black cattle.

Malhado et al. (2009) pointed out that $\beta_{1}$ is an integration constant with no specific biological meaning. In this study, the estimated $\beta_{1}$ values in the Gompertz and Richard models were 2.4064 and -0.0134 for Ayrshire cattle and 2.3610 and -0.01163 for crossbred cattle for males and females, respectively, and were lower than those obtained by Hartati and Putra (2021), who evaluated the growth curve in Madura cattle using the logistic model, obtaining values of 7.10 and 7.31 for males, and the Gompertz model, obtaining values of 2.48 and 2.51 for females. Júnior et al. (2022) determined the value of $\beta_{1}$ for female Nelore cattle using the Brody, Gompertz, and logistic models (0.9216, 2.0002, and 4.6810 in males and 0.9067, 1.9472, and 4.5291 in females, respectively). Domínguez-Viveros et al. (2023) found that the best-fitting model for estimating growth in Limousin cattle was the von Bertalanffy model, with a parameter $\beta_{1}$ of 0.5949 for males and 0.5666 for females. Sakar et al. (2023) calculated $\beta_{1}$ in Anatolian Black cattle using the Brody model (0.83) and the Gompertz model (0.40). Tutkun (2019) estimated the $\beta_{1}$ value in Holstein-Friesian bulls using the Gompertz model (3.354), the Richard model (0.299), and logistic model (13.760).

Using the Gompertz model, $\beta_{2}$ values were calculated for Ayrshire cattle: 0.006707 and 0.004153. The Richard model shows that the $\beta_{2}$ values of crossbred cattle were 0.006316 and 0.004546 for males and females, respectively. The estimated value for $\beta_{2}$ in the present study was in line with the findings of Tutkun (2019), who estimated the $\beta_{2}$ value in Holstein-Friesian bulls using different models: Gompertz – 0.004, Richard – 0.003, and logistic – 0.008. Júnior et al. (2022) determined the $\beta_{2}$ value in Nelore cattle using the Gompertz and Brody models, obtaining 0.0051 and 0.0019 for males and 0.0059 and 0.0025 for females. Domínguez-Viveros et al. (2023) estimated the $\beta_{2}$ value using the Brody, von Bertalanffy, Gompertz, and logistic models to be 0.000618, 0.00583, 0.0117, and 0.00400 for male Limousin cattle and 0.00151, 0.00656, 0.0124, and 0.0477 for female Limousin cattle. However, the $\beta_{2}$ values obtained in this study were significantly lower (0.18 and 0.09 for females and 0.19 and 0.09 for males) than those calculated by Hartati and Putra (2021) for Madura cattle using the logistic and Gompertz models.

## 5 Conclusion

This study evaluated the growth performance of Ayrshire and crossbred (F1) cattle using four non-linear growth models. While the Gompertz model sometimes provided higher explanatory power (Adj- $R^{2}$ ) and lower prediction error (OSE), the Richard model consistently yielded the lowest AIC and BIC values, making it the most reliable and parsimonious choice. Across both breeds and sexes, growth curves showed that females attained substantially higher mature body weights than males, with crossbred cattle outperforming purebred Ayrshire in terms of asymptotic weight and goodness-of-fit precision. The lower error variance observed in crossbred cattle further emphasizes the predictive accuracy of the Richard model. Overall, the Richard model provides a robust framework for describing and predicting cattle growth, offering valuable insights for genetic selection, herd management, and breeding strategies aimed at improving productivity.

## Data Availability

All raw data can be provided by the corresponding authors upon request.
